# Tuning the Magnetism in Boron-Doped Strontium Titanate

**DOI:** 10.3390/ma13245686

**Published:** 2020-12-12

**Authors:** Hui Zeng, Meng Wu, Hui-Qiong Wang, Jin-Cheng Zheng, Junyong Kang

**Affiliations:** 1 Fujian Provincial Key Laboratory of Semiconductors and Applications, Collaborative Innovation Center for Optoelectronic Semiconductors and Efficient Devices, Department of Physics, Xiamen University, Xiamen 361005, China; 19820170155498@stu.xmu.edu.cn (H.Z.); meng.wu@xmu.edu.cn (M.W.); jykang@xmu.edu.cn (J.K.); 2 Department of Physics, Xiamen University Malaysia, Sepang 43900, Selangor, Malaysia

**Keywords:** strontium titanate, boron doping, magnetism, first-principles calculations

## Abstract

The magnetic and electronic properties of boron-doped SrTiO_3_ have been studied by first-principles calculations. We found that the magnetic ground states of B-doped SrTiO_3_ strongly depended on the dopant-dopant separation distance. As the dopant–dopant distance varied, the magnetic ground states of B-doped SrTiO_3_ can have nonmagnetic, ferromagnetic or antiferromagnetic alignment. The structure with the smallest dopant-dopant separation exhibited the lowest total energy among all configurations considered and was characterized by dimer pairs due to strong attraction. Ferromagnetic coupling was observed to be stronger when the two adjacent B atoms aligned linearly along the B-Ti-B axis, which could be associated with their local bonding structures. Therefore, the symmetry of the local structure made an important contribution to the generation of a magnetic moment. Our study also demonstrated that the O-Ti-O unit was easier than the Ti-B-Ti unit to deform. The electronic properties of boron-doped SrTiO_3_ tended to show semiconducting or insulating features when the dopant–dopant distance was less than 5 Å, which changed to metallic properties when the dopant–dopant distance was beyond 5 Å. Our calculated results indicated that it is possible to manipulate the magnetism and band gap via different dopant–dopant separations.

## 1. Introduction

Doping in nonmagnetic semiconductor systems is considered to be an effective approach to attain magnetic properties. For example, ferromagnetic behavior near room temperature was observed after doping transition metal elements into wide-gap oxides in Co/Ti/Mn-doped ZnO [1,2,3], Mn-doped BaTiO_3_/SrTiO_3_/KTaO_3_ [4], Cr/Fe/Co-doped TiO_2_ [5,6,7,8], and Mn-doped SnO monolayers [9]. Further analysis indicated that nonmetallic dopants also played important roles in inducing magnetism [10,11,12,13,14]. For instance, density functional theory (DFT) calculations demonstrated that substitutional B anion and B cation doping produced total spin magnetic moments of 1.0 μ_B_ [15] and 0.76 μ_B_ [16] for anatase TiO_2_ and a B-doped ZnO monolayer (Zn_36_O_35_B), respectively. Peng et al. revealed that the calculated total magnetic moment per dopant in μ_B_ equaled the difference in the atomic number between the foreign atoms and the host anion atoms in the non-transition-metal-doped semiconductors AlN and ZnO modeled by a 3 × 3 × 2 wurtzite supercell with 72 atoms, where N was substituted by Be, B or C and O was substituted by B, C or N, the calculated total magnetic moments were 2 μ_B_ when B replaced N in AlN and 3 μ_B_ when B replaced O in ZnO [12].

As one of the most famous perovskite ceramic materials, strontium titanate (SrTiO_3_) has attracted a great deal of attention and led to promising technological applications [17,18,19,20]. Great efforts have been made to modify the electronic structures of monodoped or codoped SrTiO_3_ [21,22,23,24,25,26,27,28,29,30,31,32,33,34], e.g., to enhance the visible light photocatalytic activity or for applications in water splitting [35,36,37,38,39,40,41,42,43,44,45,46,47]. Some researchers have been devoted to manipulating the magnetic properties by doping transition metals into SrTiO_3_ both theoretically and experimentally [48,49,50,51,52]. For example, SrTiO_3_ remained paramagnetic down to low temperatures when Mn was substituted for Ti [53,54], while ferromagnetic coupling behavior appeared when Cr replaced Ti [55]. However, relatively limited studies have been devoted to making SrTiO_3_ a magnetic material by replacing oxygen with anion dopants. Experiments confirmed that B and Fe were effectively doped into the SrTiO_3_ matrix, where B substituted O anions, while Fe substituted Ti cations [38]. In one study case, it was found by all-electron calculations with a 40-atom supercell that SrTiO_2.875_B_0.125_, SrTiO_2.875_C_0.125_, and SrTiO_2.875_N_0.125_ exhibited magnetic moments of 3, 2 and 1 μ_B_, respectively [56]. Similar magnetic results were observed for the C-doped SrO-terminated SrTiO_3_ (001) surface and in different SrTiO_2.75_N_0.25_ configurations [57,58,59]. In addition, spin-polarized calculations within the framework of DFT+U demonstrated that the computed magnetic moment was 1.95 μ_B_ in the mono-boron-doped SrTiO_3_ material with a 135-atom supercell [27]. However, to the best of our knowledge, dopant–dopant magnetic coupling for boron in SrTiO_3_ is still poorly understood. Therefore, establishing and providing a good understanding of boron–boron coupling in SrTiO_3_ is essential. On the other hand, dimer pairs or clusters were reported due to the strong attraction in boron-doped or carbon-doped systems when the two dopants were nearest neighbors, which seemed to be a common phenomenon in some B-doped or C-doped systems. For instance, the boron–boron bond length was very close to the typical bond length of a boron cluster (1.48–1.70 Å), which was widely reported in TiO_2_ [15], silicon [60], and ZnO monolayers [16]. Similar results were also found in the case of C-doped ZnO monolayer [16] and TiO_2_ [13]. Nevertheless, it was difficult to generate clusters in N-N coupling configurations [58,59,61]. For the case of boron-doped SrTiO_3_, it would be interesting to investigate whether and how the formation of boron clusters depends on the boron–boron separation.

To explore the magnetic properties in boron-doped SrTiO_3_, as well as to address the underlying magnetic mechanism of boron–boron coupling, we employed DFT to simulate seven structures with different dopant–dopant separations in SrTiO_2.75_B_0.25_. The study of boron coupling in SrTiO_2.75_B_0.25_ systems may provide some theoretical guidance for designing new magnetic materials.

## 2. Computational Details

In the present study, the Vienna ab initio Simulation Package (VASP) [62,63] based on DFT [64] with projected augmented wave (PAW) potentials was employed to perform first-principles calculations [65]. The exchange-correlation functional was adopted with the generalized gradient approximation (GGA) parameterized by Perdew-Burke-Ernzerhof (PBE). The corresponding PAW potentials for valence electron sets were 4s^2^4p^6^5s^2^ for Sr, 3s^2^3p^6^3d^2^4s^2^ for Ti, 2s^2^2p^4^ for O and 2s^2^2p^1^ for B. The cutoff energy for the plane-wave basis set was 450 eV, and a 5 × 5 × 5 k-point grid centered at the Gamma (or Г) point was used. The energy convergence criterion for self-consistent iteration was set to 10^−5^ eV, and all the atomic positions were fully relaxed. The relaxation stopped if all components of the residual forces were less than 0.01 eV/Å. Electronic structures were calculated for the corresponding optimized crystal geometries. We adopted the tetrahedron method for the calculation of the electronic density of states (DOS).

**Figure 1:**
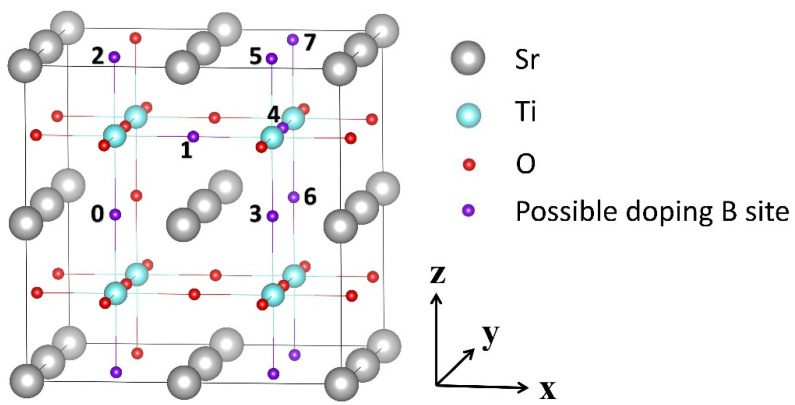
The model of the corresponding different substitutional B anion structures of SrTiO_3_ is provided. We label different configurations as (0,j), where the position of the first dopant is fixed and labeled 0, while the other alternative doping positions are labeled j. Color codes: gray for Sr, red for O atoms, cyan for Ti, and violet 0–7 for the possible positions of the B dopant. The x, y, and z axes refer to the crystallographic *a*, *b*, and *c* directions, respectively.

The calculated lattice constant of the bulk SrTiO_3_ is *a*_0_ = 3.935 Å, in good agreement with the experimental value (3.905 Å). The supercell constructed by 2 × 2 × 2 SrTiO_3_ unit-cell containing 40 atoms was used for doped system. As shown in Figure 1, seven different doping configurations were established, where two O atoms were substituted by two B atoms and separated by the nearest distance of $\sqrt{2}a_{0}/2$ to the farthest distance of $\sqrt{3}a_{0}$ (i.e., 2.782 Å to 6.816 Å) before relaxation. The position of one doped boron atom (indicated as the first dopant in the supercell) was fixed and labeled 0, while the other boron atoms (e.g., second dopant in the supercell) were labeled 1–7 with increasing separation from 0. For simplicity, these configurations were labeled as (0,j), i.e., (0,1), (0,2), (0,3), (0,4), (0,5), (0,6), and (0,7). The effects of the separation distance between dopants on the properties were investigated in detail.

## 3. Results and Discussion

### 3.1. Magnetic Property

**Figure 2:**
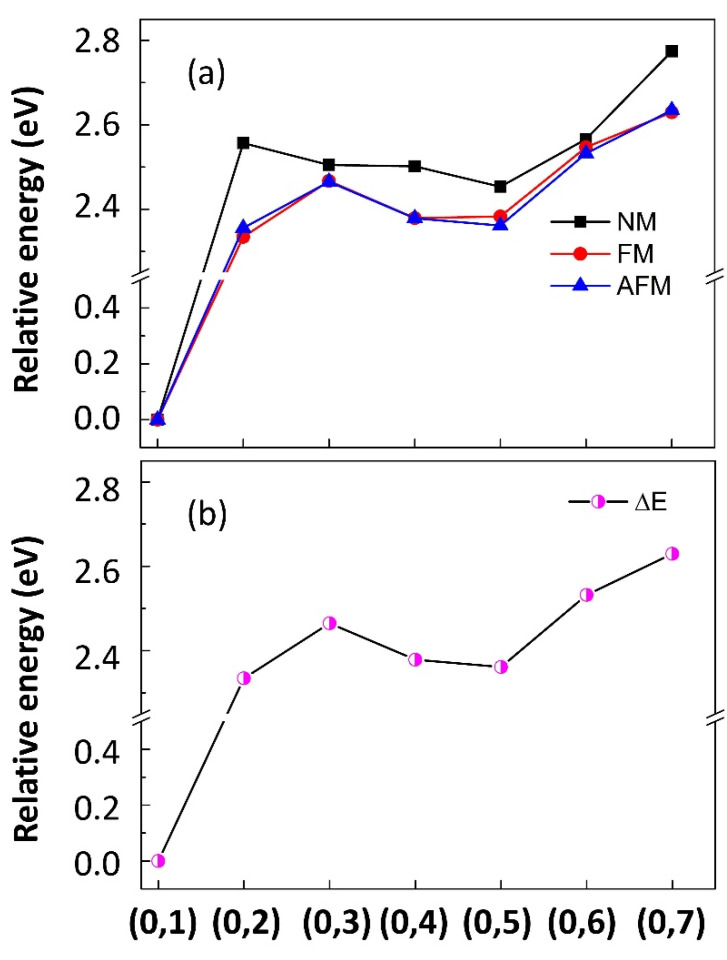
Panel (**a**): Calculated relative energy (eV/dopant) in nonmagnetic (NM), ferromagnetic (FM) and antiferromagnetic (AFM) states. Panel (**b**): Relative energy (eV/dopant) of different (0,j) structures for the B doped SrTiO_3_ system. The lowest energy is set as a reference, i.e., ∆E = E (0,j) − E (0,1) for each structure.

To gain insight into the relative stability of the different dopant configurations shown in Figure 1, under nonmagnetic (NM), ferromagnetic (FM), and antiferromagnetic (AFM) states, we performed spin-polarized calculations and addressed the detailed distinctions in energy. The results of the relative energy (eV) in different states versus different (0,j) structures are presented in Figure 2a, which is important to evaluate the coupling strength of substitutional dopants. Rather higher energies are observed under NM states compared to FM or AFM states, indicating that the latter are more favorable alignments in all configurations except for the (0,1) structure. The initial magnetic FM and AFM states of the (0,1) structure both converge to NM states, suggesting an NM nature of the ground magnetic state (GMS). To further explore the relationship in energy between the FM and AFM alignments, the relative energy (∆E, in units of eV/dopant) is employed, i.e., ∆E = E (0,j) − E (0,1) for each structure, where E (0,j) is the smaller total energy between the FM and AFM states of each structure, and the NM state of the (0,1) structure with the lowest energy among all configurations is set as a reference. An overview of the relative energy ∆E is provided in Figure 2b, and the detailed data are summarized in Table 1. Our calculated results indicate that the (0,1) structure is characterized by an approximately 2.3–2.6 eV lower energy (eV/dopant) than the other configurations, which implies that it is the most stable one. Meanwhile, the highest total energy for the (0,7) structure demonstrates that it is the most unstable one.

The distance between the two B dopants before relaxation varies from the nearest distance of $\sqrt{2}a_{0}/2$ to the farthest distance of $\sqrt{3}a_{0}$ (i.e., 2.782 Å to 6.816 Å), corresponding to configurations (0,1) to (0,7), where a_0_ is the lattice constant of bulk SrTiO_3_. Among them, the (0,2) and (0,3) structures have the same dopant-dopant separation distances of a_0_ (i.e., 3.935 Å), while the (0,5) and (0,6) structures have the same dopant-dopant separation distances of $\sqrt{2}a_{0}$ (i.e., 5.565 Å), respectively. As seen from Table 1, the dopant–dopant distance (R_d_) in the (0,1) structure significantly decreases to 1.513 Å after relaxation, which implies that the strong attraction between boron atoms induces a dimer pair, as the bond length of B-B is comparable to the typical bond length of a boron cluster (1.48–1.70 Å). For instance, clustering of boron has been widely reported in TiO_2_ [15], silicon [60], and ZnO monolayers [16]. Similar clustering of dopants was also found in the case of the C-doped ZnO monolayer [16] and TiO_2_ [13], which indicated that it might be a general trend for dopants to form a cluster.

However, although it is theoretically possible to form all kinds of clusters, when comparing a variety of configurations with different dopant–dopant distance, usually it is those configurations with lower relative energy that tends to form. It was found that the two B dopants in anatase TiO_2_ could easily form an interstitial B-like structure [15], which was also verified by experiments [66]. The Similar result was shown for two neighboring C or B atoms in a ZnO monolayer forming a dimer structure with an NM ground state, and the formation energy was found to decrease as the two doped C or B atoms became closer [16].

For B doped-SrTiO_3_, the doping structure with a nonmagnetic feature is most stable when the boron dopants are nearest neighbors in structure (0,1) (as shown in Table 1), which could be responsible for the formation of the B-B dimer pair. Stronger B-B interactions versus B-Ti hybridization in this structure are verified by the band structures, which will be discussed in detail below. The energy differences between the FM and AFM states (E_MM_, in units of eV/dopant) for each structure, i.e., E_MM_ = E_FM_ − E_AFM_, the relevant nature of the ground magnetic state (GMS), are listed in Table 1. For the (0,2) structure, the FM state is more stable than the AFM state by 0.021 eV, which demonstrates a preference for the FM state. Although the FM state is energetically favored for the (0,7) structure, or AFM for the (0,3), (0,5) and (0,6) configurations, their absolute values of E_MM_ are relatively small, e.g., less than 25 meV, and both FM and AFM are labeled as possible GMSs taking into account the temperature effects and numerical uncertainty. In addition, for the (0,4) magnetic coupling structure, the magnetic energy difference becomes almost negligible. Therefore, as the dopant–dopant distance decreases, the GMSs of different structures exhibits a variety of magnetic features, ranging from FM/AFM to NM alignment.

To further explore the magnetic coupling difference under FM and AFM alignments for different (0,j) structures in B-doped SrTiO_3_, we provide the calculated total magnetic moment (MM_tot_), localized magnetic moment on boron (MM_B_), and averaged contribution to the MM for the Ti (MM_Ti_) atom in units of μ_B_, as shown in Table 1. Except for the (0,2) configuration, the total magnetic moment in FM states increases from 0 to 4.40 μ_B_ with increasing dopant–dopant separation due to the increasingly weaker interaction of the dopants. Therefore, the magnetic properties of B-doped SrTiO_3_ strongly depend on the distance between boron dopants. For the (0,2) case, the two boron atoms align in a linear way along the B-Ti-B axis, and one can obtain a considerable magnetic moment of 3.94 μ_B_. Additionally, although the (0,2) and (0,3) structures are characterized by the same dopant–dopant distance before relaxation, they exhibit completely different MM_tot_. Different magnetic moments have also been reported in the literature, e.g., all configurations acquired almost equal magnetic moments of approximately 2 μ_B_ for SrTiO_2.75_N_0.25_ [58], while MM_tot_ remained at 0 μ_B_ or was approximately 2.0 μ_B_ for all structures in two-B-atom-doped anatase TiO_2_ [15].

The calculated total magnetic moment of 4.40 μ_B_ for the (0,7) structure is worth further investigation in view of the relatively large distance between dopants. First, the calculated magnetic moment per dopant in μ_B_ has been ascribed to the atomic number difference between the dopant and the host anion atom based on theoretical calculations for AlN and ZnO semiconductors [12]. Generally, one N replaces O, introducing one hole and generating a total magnetic moment of approximately 1 μ_B_. Similarly, one C(B)-substituted O will introduce two (three) holes and give rise to a total magnetic moment of approximately 2 (3) μ_B_. Therefore, the two-nitrogen-doped system replacing O introduces a magnetic moment of approximately 2 μ_B_ when the two dopants are far apart, accompanying small N-N coupling, and the two-carbon (boron)-doped system generates a total magnetic moment of approximately 4 (6) μ_B_, as reported in SrTiO_3_, TiO_2_, ZnO and other systems. For example, Bannikov et al. mentioned that the magnetic moment obtained for B-doped SrTiO_3_ was 3 μ_B_ [56]. However, the total magnetic moment obtained by DFT+U calculations was 1.95 μ_B_ [27]. To explore this discrepancy, as well as to understand why the magnetic moment in the (0,7) structure with the weakest B-B coupling we calculated is approximately 4 μ_B_ rather than 6 μ_B_, we performed single B/C/N doping in SrTiO_3_ to repeat the previous work. We found that although we obtain the same total magnetic moment as that of N(C)-doped SrTiO_3_ in the literature, the total moment of B-doped SrTiO_3_ is approximately 2 μ_B_, i.e., the total magnetic moment of SrTiO_3_ doped with N, C, and B is 0.98, 2.00, 2.08 μ_B_, whereas the magnetic moment obtained by Bannikov et al. was 1.00, 2.02, and 3.00 μ_B_, respectively. We further performed fixed-spin-moment calculations for boron-doped SrTiO_3_, which shows fixed magnetic moments of 1, 2, and 3 μ_B_ versus relative energies of 73.1, 0, and 29.7 meV (the state of 2.08 μ_B_ MM has a lower energy by approximately 4 meV than that of 2 μ_B_ MM), confirming the total MM of 2.08 μ_B_ with respect to the lowest energy. The partial density of states (PDOS) of the introduced B ion (Supplemental Materials Figure S1) shows that most of the up-spin B 2p_x_, 2p_y_ and 2p_z_ orbitals and approximately half of the down-spin B 2p_x_ orbital are occupied, while the other B 2p orbitals are unoccupied, which illustrates that the doped B ion in SrTiO_3_ may have the electron configuration of s^2^p^3^(B^2−^), thus leading to a net magnetic moment of 2.08 μ_B_. Moreover, the result obtained for our B-doped SrTiO_3_ is close to the result by Maldonado et al. [27]. In other words, each B dopant introduces a magnetic moment of 2.08 μ_B_, and a total magnetic moment of 4.40 μ_B_ is expected in the boron-doped SrTiO_3_ system. In fact, a similar discrepancy also appeared in the B-B-coupled TiO_2_ system, in which the authors found that the total magnetic moment was equal to approximately 1 μ_B_, rather than 3 μ_B_ per dopant, which was ascribed to the different symmetries [15]. Different local structures correspond to different symmetries and produce different electronic occupied states, which in turn cause different magnetic moments, e.g., BTi_2_(SrTiO_3_), BTi_3_(TiO_2_), and BZn_4_(ZnO) have total MMs of 2.08, 1, and 3 μ_B_, respectively. Therefore, in addition to the atomic number difference between the dopant and the host anion, the symmetry of the local structure makes an important contribution to the generation of a spin-polarized magnetic moment.

Table 1 shows that the resolved magnetic moment qualitatively originates mainly from the impurity boron atoms, relatively small localized magnetic moments are detected at the Ti or O atoms adjacent to the dopants, and no noticeable contribution to the magnetic moments is observed at the Sr atoms (Supplemental Materials Table S1). We note that the total magnetic moment is less than the sum of the magnetic moments of each atom, which is ascribed to the default radius set for integration of the magnetic moment. This does not affect our conclusions, especially the trends we will discuss in the following sections, with the aim of investigating the underlying coupling mechanisms of the observed peculiarity with respect to different FM (0,j) alignments.

### 3.2. Structure

As discussed above, the optimized dopant–dopant distance ranges from 1.513 Å to 6.895 Å; therefore, there is a remarkable displacement of dopant–dopant distance after relaxation compared to un-doped SrTiO_3_ for the (0,1) structure, which can be attributed to the strong attraction between boron atoms. Together with the considerable shift of B-Ti, the two remarkable variations in the structure of configuration (0,1) render the energetically favored NM GMS feature. As shown in Table 2, the values of ∆(R_d_) and ∆(B-Ti) in all other configurations accompanying FM or AFM alignment are less than 0.2 Å, which demonstrates a weaker attraction or repulsion. Configurations (0,3) and (0,4) show a tiny B-B attraction, while the other configurations except for the (0,1) arrangement exhibit repulsion between the dopants. Ti atoms tend to move away from the nearest boron atom in all cases.

The positive values of ∆*a,* ∆*b,* and ∆*c* in most cases indicate a larger volume, which is partly ascribed to the larger ionic radius of the B dopants compared to O ions. As seen from Table 2, even with great changes in the relative distances of R_d_ and B-Ti (Å), a remarkable variation in the lattice constants is not generated, e.g., in the (0,1) structure. Moreover, the replacement of the host O by the foreign B atoms seems to easily break the original symmetry of the oxygen octahedron and induce crystal field splitting. Therefore, a certain distortion or twist can also be observed. The produced distortion or twist of the oxygen octahedron has a great influence on the magnetic properties of the material. Thus, investigating the interactions between the angles and MM in different (0,j) alignments can contribute to a better understanding of the underlying coupling mechanisms. Before relaxation, the bond angles of Ti-B-Ti and O-Ti-O align linearly, i.e., ∠Ti-B-Ti = ∠O-Ti-O = 180°. The interrelated relaxation results for different (0,j) configurations are presented in Table 2. One can see that ∠Ti-B-Ti and ∠O-i-O are equal to 137.884° and 170.935° in the (0,1) structure, respectively, i.e., distinct bending of the Ti-B-Ti and O-Ti-O units occurs, which is relevant to an NM character. However, the relatively large MM (3.94 μ_B_) in the (0,2) configuration can correspond to or be responsible for the invariable Ti-B-Ti and O-Ti-O units.

**Figure 3:**
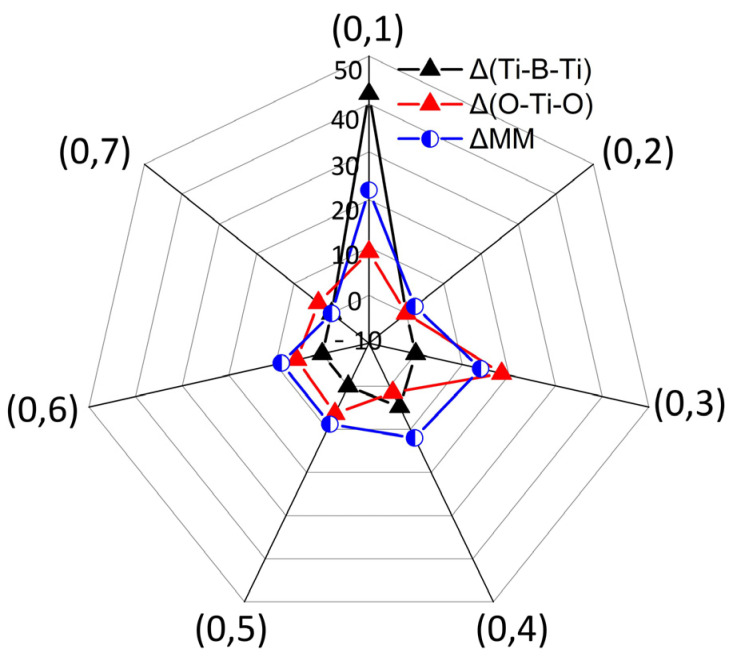
The relationship among bent Ti-B-Ti bond angle, O-Ti-O bond angle and change in MM (in the FM state), i.e., ∆(Ti-B-Ti), ∆(O-Ti-O), and ∆MM, for different (0,j) structure, where **∠**Ti-B-Ti = **∠**O-Ti-O = 180° and the maximum MM (4.40 μ_B_) under FM alignment among all configurations are taken as references. The values of ∆MM are amplified five times for clarity.

**Figure 4:**
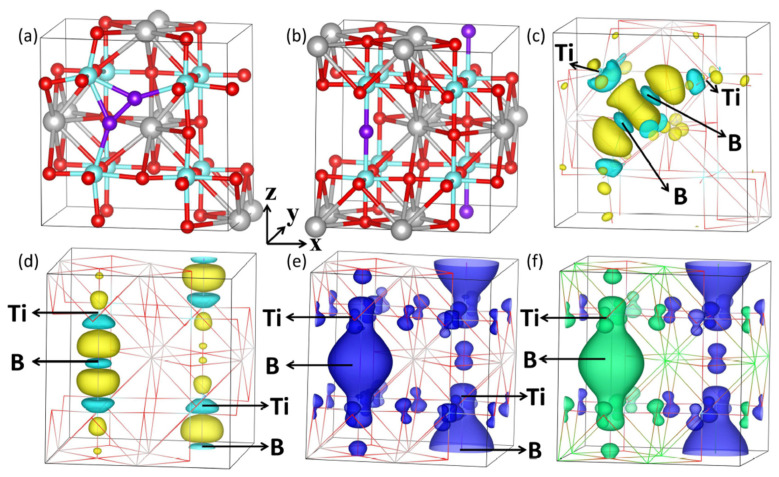
Relaxed structures of configurations (0,1) and (0,7) in panels (**a**) and (**b**), respectively. The gray, red, cyan, and violet spheres in panels (**a**,**b**) correspond to the Sr, O, Ti and B atoms, respectively. The corresponding charge density differences are shown in (**c**) and (**d**). The yellow and violet–red areas represent charge accumulation and charge depletion, respectively. Spin densities under (**e**) FM alignment and (**f**) AFM alignment for structure (0,7). The blue and green areas represent majority and minority spin densities, respectively. The isosurface level value is set as 0.005 e/Å^3^ for the calculations of charge density differences and spin densities.

We provide the interactions among ∆(Ti-B-Ti), ∆(O-Ti-O), and ∆MM in different (0,j) arrangements to explore the underlying coupling mechanisms, as shown in Figure 3. The values of 180° for the Ti-B-Ti and O-Ti-O bond angles and 4.40 μ_B_ for the MM are employed as references. Figure 3 shows that enormous angular changes ∆(Ti-B-Ti) and ∆(O-Ti-O) are observed for the (0,1) structure, especially the greater bent Ti-B-Ti bond angle with respect to the O-Ti-O bond angle, i.e., approximately 42° versus 9°. The structural changes are ascribed to the formation of the B-B dimer, as shown in Figure 4a, which finally give rise to the most energetically favored state in all (0,j) configurations and converge to NM alignment. The relatively large MM in the (0,2) configuration is associated with the invariable Ti-B-Ti and O-Ti-O units. For the other configurations, except for a slight bending (4.5°) in the (0,4) structure, the Ti-B-Ti bond angle remains basically unchanged. However, the O-Ti-O bond angle is variable in the five configurations from 3.8° to 18.5°, which implies that the O-Ti-O unit is easier to deform. For clarity, we magnified the MM in the FM state five times. As shown in Figure 3, we can speculate that the bending of bond angles is basically positively correlated with ∆MM. For example, for the invariable Ti-B-Ti unit in the (0,3) and (0,5) structures, a larger ∆(O-Ti-O) gives rise to a larger ∆MM. Remarkable bending of the O-Ti-O bond angles and ∆MM are visible in the (0,3) configuration. The slightly 4.5° bent Ti-B-Ti unit and 1.7° bent O-Ti-O unit in the (0,4) structure with respect to the fixed Ti-B-Ti unit and the 18.5° bent O-Ti-O unit in the (0,3) structure trigger ∆MM values of 12.01 μ_B_ and 13.97 μ_B_ for the two configurations respectively, which indicates that the magnetism in different (0,j) configurations is theoretically associated with their structures, e.g., correlated with the bending of Ti-B-Ti and O-Ti-O units.

Calculations of the charge density difference, Bader charge, and spin density were further performed in this work. For comparison, we chose the most stable and unstable structures, i.e., the (0,1) and (0,7) configurations. In accordance with the above discussion, as shown in Figure 4, the two impurity boron atoms tend to strongly attract each other and form a B-B dimer pair in structure (0,1). To understand the charge transformation and redistribution, the charge density differences around B atoms of the (0,1) and (0,7) configurations are shown in Figure 4c,d, respectively. Herein, the isosurface level value is set as 0.005 e/Å^3^, and the yellow areas represent charge accumulation, while violet–red areas represent charge depletion. The variation in the electronic distribution with charge transfer to the boron is rather obvious. The significant charge accumulation near the boron and small amount of charge depletion around the titanium atoms closest to the boron in both the (0,1) and (0,7) structures give rise to strong electrostatic interactions between the titanium and boron atoms. As shown in Supplemental Materials Figure S2, other charge density differences are characterized by similar properties. Table 2 shows the charge transfer to the two doped boron atoms in all configurations based on Bader charge analysis. In the (0,1) structure, the two Boron dopants acquire 0.30 e and 1.34 e charges, respectively, while in the (0,7) structure, they acquire 0.71 e and 0.71 e charges, respectively; i.e., each B atom gains an average of 0.82 e and 0.71 e charge for the two structures, respectively.

Next, we focus on the magnetic properties of the B dopants in SrTiO_3_. To understand the origin of the magnetic moment, the spin densities under FM alignment (e) and AFM alignment (f) in structure (0,7) are presented. The blue and green areas represent majority and minority spin densities, respectively. We can clearly observe from Figure 4e that most of the MM under the FM state is qualitatively attributable to the B atoms, relatively small localized MMs are visible at the Ti or O atoms, and no noticeable contribution to the MM is related to the Sr atoms, in agreement with our previous discussions. Moreover, the parallel orientation for all atoms under FM alignment in the (0,7) structure gives rise to a remarkable MM.

### 3.3. Electronic Structure

**Figure 5:**
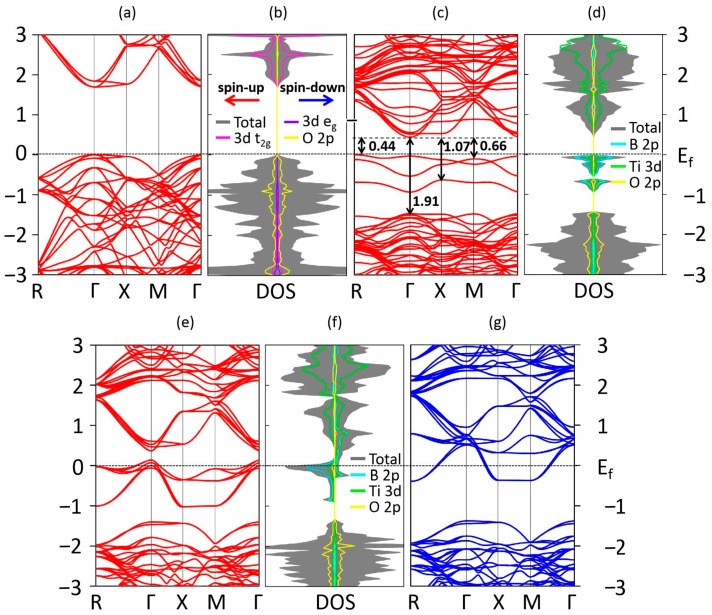
Calculated (**a**) band structure (spin-up channel), (**b**) total density of states (DOS) and partial density of states (PDOS) for the Ti 3d t_2g_, Ti 3d e_g_, and O 2p orbitals of un-doped SrTiO_3_. (**c**) Band structure (spin-up channel), (**d**) DOS and PDOS for the B 2p, Ti 3d and O 2p orbitals of the (0,1) structure. (**e**) Band structure (spin-up channel), (**f**) DOS and PDOS for the B 2p, Ti 3d and O 2p orbitals and (**g**) band structure (spin-down channel) of the (0,7) structure. The Fermi level is marked by a dashed line. PDOS plots are all amplified five times for clarity.

Figure 5a,b shows the calculated band structure, total density of states (DOS) and partial density of states (PDOS) for the Ti 3d t_2g_, Ti 3d e_g_ and O 2p orbitals of un-doped SrTiO_3_. Our calculated band gap is 1.69 eV, which is less than the experimental value of 3.20 eV due to the typical underestimated calculation by local density approximation (LDA) or GGA [67,68]. The valence bands and conduction bands of un-doped SrTiO_3_ are predominantly composed of O 2p orbitals and Ti 3d orbitals, respectively. For the unoccupied conduction bands, three lower energy t_2g_ orbitals (3d_xy_, 3d_yz_, 3d_zx_) and two higher energy e_g_ orbitals (3d_z_^2^, 3d_x_^2^_− y_^2^) are observed, which are ascribed to the splitting of the crystal field in the TiO_6_ octahedral structure. The symmetric spin-up and spin-down channels presented in Figure 5b demonstrate that un-doped SrTiO_3_ exhibits a nonmagnetic property. Based on the electronic features, the (0,1), (0,2), and (0,4) structures exhibit a semiconductor or insulator character, while metal properties are shown for the other four configurations. We choose to further explore the most stable and unstable structures, i.e., the (0,1) and (0,7) configurations, to gain insights into the electronic properties.

The DOS and PDOS for the B 2p, Ti 3d and O 2p orbitals of the (0,1) structure are shown in Figure 5d. The equal numbers of spin-up and spin-down electrons give rise to an NM ground state character, consistent with our magnetic studies above. Moreover, it is obvious that both the spin-up and spin-down channels do not cross the Fermi level, which suggests that the (0,1) configuration still retains a semiconducting or insulating property. Figure 5c presents the electronic bands of configuration (0,1). One can observe that three distinct isolated occupied states composed of the foremost B 2p, weak Ti 3d and O 2p orbitals are localized above the top of the original valence band but below the Fermi level, becoming the new valance band maximum (VBM). The stronger B-B interactions compared to the C-Ti hybridization near the Fermi level in the SrTiO_2.75_B_0.25_ system correspond to the nonmagnetic ground state feature. Two extra bands dominantly composed of the Ti 3d and B 2p orbitals become the new conduction band minimum (CBM). The positions of the B orbitals localized between the original VBM and CBM are ascribed to the higher orbital energies of the foreign B 2p orbitals than those of the host O 2p orbitals. In addition, the Sr atoms have almost no contribution to the electronic structure; therefore, this contribution is not shown here.

In our calculations for the (0,1) structure, as shown in Figure 5c,d, the hybridization of the B 2p orbitals and Ti 3d orbitals pulls the CBM downward to the position of 0.44 eV above the Fermi level, while compelling the original VBM to be located at approximately 1.47 eV below the Fermi level, providing a band gap of 1.91 eV. Therefore, the bands originating from B 2p states lead to widening instead of narrowing of the band gap. The energies from the three distinct isolated occupied states to the CBM are 0.44, 0.66, and 1.07 eV, which are smaller than the energies of un-doped SrTiO_3_ by 1.25, 1.03, and 0.62 eV, respectively. The induced isolated energy levels may contribute to the enhancement of the photocatalytic activity under visible light, even though the uncompensated dopants play a significant role as recombination centers and are detrimental to the separation and migration of photogenerated electron-hole pairs.

The calculated band structures (spin-up and spin-down channels) and spin-polarized DOS of the (0,7) structure are shown in Figure 5e,f,g, where the uncompensated spin-up and spin-down channels illustrate the existence of a magnetic moment. Meanwhile, the Fermi level crosses both the spin-up and spin-down channels, which indicates that the (0,7) configuration has a metallic nature. Figure 5f shows that the asymmetric populations are generated from the valance band near the Fermi level, as well as from the conduction band, i.e., the contributions to the MM come from both occupied and unoccupied states. On the other hand, more hybridization between the B 2p and Ti 3d orbitals near the Fermi level is observed in the spin-up channel. These hybridized orbitals are separated by the original VBM and CBM; thus, the in-gap states actually have almost no contribution to the narrowing of the band gap. Therefore, for the spin-up channel, the hybridization leads to the CBM position of 0.36 eV above the Fermi level, while the original VBM is located at approximately 1.40 eV below the Fermi level, giving rise to a band gap of 1.76 eV. For the spin-down channel of the (0,7) configuration, more holes are induced compared to the spin-up channel. In addition, the orbitals of the dopants overlap with the Ti 3d orbitals, which increases the width of the CB and reduces the band gap. In our calculation, the CBM is downshifted to approximately 0.39 eV below the Fermi level, while the VBM is localized at approximately 1.41 eV below the Fermi level, which finally gives rise to a band gap of 1.02 eV and produces a significant band narrowing of approximately 0.67 eV.

### 3.4. Conclusions

We performed DFT with the GGA method to explore the magnetic and electronic coupling properties of boron-doped SrTiO_3_ within the framework of different dopant–dopant separations. We found that the GMS of B-doped SrTiO_3_ strongly depends on the dopant–dopant separation distance, i.e., the GMS of different structures transforms from FM/AFM and subsequently converges to NM alignment as the distance of the two doped boron atoms decreases. When the two doped boron atoms have the smallest dopant–dopant separation distance, the structure is characterized by the lowest energy among all structures, and a dimer pair is visible. Strong FM coupling is observed when two doped boron atoms align linearly along the B-Ti-B axis, which can be theoretically associated with the structure, e.g., correlated with the bending of Ti-B-Ti and O-Ti-O units. Therefore, the symmetry of the local structure makes an important contribution to the generation of a spin-polarized magnetic moment. Our study also demonstrates that the O-Ti-O unit is easier to deform than the Ti-B-Ti unit.

When the dopant–dopant distance is less than 5 Å, a semiconductor or insulator electronic structural character tends to be generated, while metal properties are present for the dopant–dopant distances beyond 5 Å. The bands originating from B 2p states lead to widening instead of narrowing of the band gap when the two doped boron atoms are nearest neighbors, even though the induced isolated energy levels can somehow contribute to enhancement of the photocatalytic activity under visible light. However, when the two doped boron atoms are the farthest, a significant band narrowing of approximately 0.67 eV is triggered, introducing more holes for the spin-down channel than the spin-up channel. Therefore, the calculated results indicate that we can theoretically manipulate the magnetism and band gap via different B-B dopant distances.

## Figures and Tables

**Table 1:** Relaxed distance between two foreign B atoms (R_d_ (Å)), relative energy (∆E, in units of eV/dopant), energy difference between FM and AFM states (E_MM_, in units of eV/dopant), total magnetic moment (MM_tot_), localized magnetic moment on boron (MM_B_), and average contribution to the MM for the Ti (MM_Ti_) atom in units of μ_B_ for different (0,j) structures in B-doped SrTiO_3_ under FM and AFM alignments. The lowest total energy of (0,1) structure is taken as a reference for the calculation of relative energy ∆E. For each (0,j) structure, the energy of the FM state is given with respect to that of the AFM state in units of eV/dopant, i.e., E_MM_ = E (FM) − E (AFM). The data in bold font highlight the nature of the ground magnetic state (GMS) for each configuration. (Note: If the energy difference between the FM and AFM states is relatively small, e.g., less than the typical value of 25 meV, then both the FM and AFM states are labeled as possible GMSs (to take into account the temperature effects and numerical uncertainty)).

(0,j)	GMS	R_d_	∆E	E_MM_	MM_tot_	MM_B_	MM_Ti_
(0,1)	NM	1.513	0	~0	0	0	0
(0,2)	FM	4.026	2.335	−0.021	3.94	0.51, 0.51	0.09
	AFM	4.023	2.356	-	0	0.55, −0.55	0
(0,3)	FM	3.896	2.467	0.002	1.61	0.24, 0.24	0.03
	AFM	3.896	2.465	-	0	0.24, −0.24	0
(0,4)	FM	4.791	2.379	~0	2.00	0.28, 0.28	0.03
	AFM	4.790	2.379	-	0	0.28, −0.28	0
(0,5)	FM	5.650	2.383	0.022	2.64	0.37, 0.37	0.09
	AFM	5.649	2.361	-	0	0.48, −0.48	0
(0,6)	FM	5.571	2.547	0.015	2.64	0.38, 0.37	0.07
	AFM	5.572	2.532	-	0.01	0.47, −0.47	0
(0,7)	FM	6.895	2.630	−0.005	4.40	0.60, 0.60	0.16
	AFM	6.895	2.635	-	0	0.60, 0.60	0

**Table 2:** ∆(R_d_) and ∆(B-Ti) represent the changes in distance of two boron dopants and B-Ti (the Ti adjacent to B, in units of Å) compared to those of un-doped SrTiO_3_ with a 2 × 2 × 2 supercell, respectively. Negative and positive values correspond to attraction and repulsion, respectively. Parameters ∆*a*, ∆*b*, ∆*c*, and the angles ∠Ti-B-Ti and ∠O-Ti-O (°) represent the variation in lattice constants (Å) and angles for different structures with respect to the lattice parameters of the un-doped SrTiO_3_ supercell. The charge (e) denotes the charge transfer to the two doped boron atoms.

(0,j)		∆(R_d_)	∆(B-Ti)	∆*a*	∆*b*	∆*c*	∠Ti-B-Ti	∠O-Ti-O	Charge
(0,1)		−1.269	0.235	0.093	0.093	0.045	137.884	170.935	(0.30, 1.34)
(0,2)	FM	0.091	0.046	0.022	0.183	0.022	180.000	180.000	(0.63, 0.63)
	AFM	0.088	0.044	0.022	0.177	0.022	180.000	180.000	
(0,3)	FM	−0.039	0.142	−0.078	0.262	0.080	180.000	161.546	
	AFM	−0.039	0.143	−0.078	0.262	0.080	180.000	161.641	(0.59, 0.59)
(0,4)	FM	−0.028	0.177	0.185	0.185	−0.032	175.211	178.560	
	AFM	−0.029	0.179	0.184	0.184	−0.031	175.460	178.306	(0.67, 0.67)
(0,5)	FM	0.085	0.170	−0.007	0.245	0.041	180.000	173.640	
	AFM	0.084	0.169	−0.006	0.241	0.041	180.000	173.661	(0.72, 0.72)
(0,6)	FM	0.006	0.169	0.008	0.249	0.008	180.000	174.541	
	AFM	0.007	0.166	0.010	0.240	0.010	180.000	176.494	(0.74, 0.74)
(0,7)	FM	0.079	0.149	0.020	0.234	0.020	180.000	176.235	(0.71, 0.71)
	AFM	0.079	0.149	0.020	0.234	0.020	180.000	176.455	

## Supplementary Materials

The following are available online at https://www.mdpi.com/1996-1944/13/24/5686/s1, Figure S1: The calculated partial density of states (PDOS) of the B 2p_x_, 2p_y_, and 2p_z_ states in B-doped SrTiO_3_ (one boron in 40-atom supercell), Figure S2: (a)–(e): The charge density differences from structure (0,2) to (0,6). The yellow and violet red areas represent charge accumulation and charge depletion, respectively. The isosurface level value is set as 0.005 e/Å^3^, Table S1: Average contribution of MM for O (MM_O_) and Sr (MM_Sr_) atom in units of μ_B_ under FM and AFM alignments for different (0,j) structures in SrTiO_2.75_B_0.25_.

## References

[1] Venkatesan M., Fitzgerald C.B., Lunney J.G., Coey J.M.D. (2004). Anisotropic ferromagnetism in substituted zinc oxide. Phys. Rev. Lett..

[2] Gacic M., Jakob G., Herbort C., Adrian H. (2007). Magnetism of Co-doped ZnO thin films. Phys. Rev. B.

[3] Jung S.W., An S.-J., Yi G.-C., Jung C.U., Lee S.-I., Cho S. (2002). Ferromagnetic properties of Zn_1−x_Mn_x_O epitaxial thin films. Appl. Phys. Lett..

[4] Norton D.P., Theodoropoulou N.A., Hebard A.F., Budai J.D., Boatner L.A., Pearton S.J., Wilson R.G. (2003). Properties of Mn-Implanted BaTiO_3_, SrTiO_3_, and KTaO_3_. Electrochem. Solid-State Lett..

[5] Kim Y.J., Thevuthasan S., Droubay T., Lea A.S., Wang C.M., Shutthanandan V., Chambers S.A., Sears R.P., Taylor B., Sinkovic B. (2004). Growth and properties of molecular beam epitaxially grown ferromagnetic Fe-doped TiO_2_ rutile films on TiO_2_(110). Appl. Phys. Lett..

[6] Wang Y.X., Liu H., Li Z.Q., Zhang X.X., Zheng R.K., Ringer S.P. (2006). Role of structural defects on ferromagnetism in amorphous Cr-doped TiO_2_ films. Appl. Phys. Lett..

[7] Shutthanandan V., Thevuthasan S., Heald S.M., Droubay T., Engelhard M.H., Kaspar T.C., McCready D.E., Saraf L., Chambers S.A., Mun B.S. (2004). Room-temperature ferromagnetism in ion-implanted Co-doped TiO_2_(110) rutile. Appl. Phys. Lett..

[8] Matsumoto Y., Murakami M., Shono T., Hasegawa T., Fukumura T., Kawasaki M., Ahmet P., Chikyow T., Koshihara S., Koinuma H. (2001). Room-temperature ferromagnetism in transparent transition metal-doped titanium dioxide. Science.

[9] Han R., Yan Y. (2018). Magnetism induced by Mn atom doping in SnO monolayer. Chin. Phys. B.

[10] Gonçalves R.D., Azevedo S., Moraes F., Machado M. (2009). Electronic structure of boron nitride nanostructures doped with a carbon atom. Eur. Phys. J. B.

[11] Pan H., Feng Y.P., Wu Q.Y., Huang Z.G., Lin J. (2008). Magnetic properties of carbon doped CdS: A first-principles and Monte Carlo study. Phys. Rev. B.

[12] Peng X., Ahuja R. (2009). Non-transition-metal doped diluted magnetic semiconductors. Appl. Phys. Lett..

[13] Yang K., Dai Y., Huang B., Whangbo M.-H. (2008). On the possibility of ferromagnetism in carbon-doped anatase TiO_2_. Appl. Phys. Lett..

[14] Pan H., Yi J.B., Shen L., Wu R.Q., Yang J.H., Lin J.Y., Feng Y.P., Ding J., Van L.H., Yin J.H. (2007). Room-temperature ferromagnetism in carbon-doped ZnO. Phys. Rev. Lett..

[15] Yang K., Dai Y., Huang B. (2010). Density Functional Study of Boron-Doped Anatase TiO_2_. J. Phys. Chem. C.

[16] Guo H., Zhao Y., Lu N., Kan E., Zeng X.C., Wu X., Yang J. (2012). Tunable Magnetism in a Nonmetal-Substituted ZnO Monolayer: A First-Principles Study. J. Phys. Chem. C.

[17] Modak B., Ghosh S.K. (2015). Enhancement of Visible Light Photocatalytic Activity of SrTiO_3_: A Hybrid Density Functional Study. J. Phys. Chem. C.

[18] Zhao K.L., Chen D., Li D.X. (2010). Effects of N adsorption on the structural and electronic properties of SrTiO_3_(001) surface. Appl. Surf. Sci..

[19] Crespillo M.L., Graham J.T., Agulló-López F., Zhang Y., Weber W.J. (2017). Role of oxygen vacancies on light emission mechanisms in SrTiO_3_ induced by high-energy particles. J. Phys. D Appl. Phys..

[20] You J.H., Lee J.H., Okamoto S., Cooper V., Lee H.N. (2015). Strain effects on the electronic properties in δ-doped oxide superlattices. J. Phys. D Appl. Phys..

[21] Al-Hadidi M., Goss J.P., Briddon P.R., Al-Hamadany R., Ahmed M., Rayson M.J. (2015). Carbon impurities in SrTiO_3_ from first principles. Modell. Simul. Mater. Sci. Eng..

[22] Yoon H.J., Kim S.K., Huang W., Sohn Y. (2018). Comparable electrocatalytic performances of carbon- and Rh-loaded SrTiO_3_ nanoparticles. Chin. Chem. Lett..

[23] Iwazaki Y., Gohda Y., Tsuneyuki S. (2014). Diversity of hydrogen configuration and its roles in SrTiO_3−δ_. APL Mater..

[24] Breckenfeld E., Wilson R., Karthik J., Damodaran A.R., Cahill D.G., Martin L.W. (2012). Effect of Growth Induced (Non)Stoichiometry on the Structure, Dielectric Response, and Thermal Conductivity of SrTiO_3_ Thin Films. Chem. Mater..

[25] Liu H.F. (2012). Effect of nitrogen and carbon doping on electronic properties of SrTiO_3_. Solid State Commun..

[26] Zhang C., Jia Y., Jing Y., Yao Y., Ma J., Sun J. (2013). Effect of non-metal elements (B,C,N,F,P,S) mono-doping as anions on electronic structure of SrTiO_3_. Comput. Mater. Sci..

[27] Maldonado F., Maza L., Stashans A. (2017). Electronic properties of Cr-, B-doped and codoped SrTiO_3_. J. Phys. Chem. Solids.

[28] Ohta S., Nomura T., Ohta H., Koumoto K. (2005). High-temperature carrier transport and thermoelectric properties of heavily La- or Nb-doped SrTiO_3_ single crystals. J. Appl. Phys..

[29] Akbar W., Liaqat T., Elahi I., Zulfiqar M., Nazir S. (2020). Modulated electronic and magnetic properties of 3d TM-doped SrTiO_3_: DFT + U study. J. Magn. Magn. Mater..

[30] Okamoto J., Shimizu G., Kubo S., Yamada Y., Kitagawa H., Matsushita A., Yamada Y., Ishikawa F. (2009). Thermoelectric properties of B-doped SrTiO_3_ singe crystal. J. Phys. Conf. Ser..

[31] Liu C.M., Zu X.T., Zhou W.L. (2007). Photoluminescence of nitrogen doped SrTiO_3_. J. Phys. D Appl. Phys..

[32] Ascienzo D., Yuan H., Greenbaum S., Bayer T., Maier R., Wang J.-J., Randall C., Dickey E., Zhao H., Ren Y. (2016). Investigation of Electric Field–Induced Structural Changes at Fe-Doped SrTiO_3_ Anode Interfaces by Second Harmonic Generation. Materials.

[33] Marozau I., Shkabko A., Döbeli M., Lippert T., Logvinovich D., Mallepell M., Schneider C., Weidenkaff A., Wokaun A. (2009). Optical Properties of Nitrogen-Substituted Strontium Titanate Thin Films Prepared by Pulsed Laser Deposition. Materials.

[34] Miruszewski T., Dzierzgowski K., Winiarz P., Wachowski S., Mielewczyk-Gryń A., Gazda M. (2020). Structural Properties and Water Uptake of SrTi_1−x_FexO_3−x_/_2−δ_. Materials.

[35] Wei W., Dai Y., Guo M., Yu L., Jin H., Han S., Huang B. (2010). Codoping synergistic effects in N-doped SrTiO_3_ for higher energy conversion efficiency. Phys. Chem. Chem. Phys..

[36] Liu J., Wang L., Liu J., Wang T., Qu W., Li Z. (2009). DFT study on electronic structures and optical absorption properties of C, S cation- doped SrTiO_3_. Cent. Eur. J. Phys..

[37] Li N., Yao K.L. (2012). The electronic and optical properties of carbon-doped SrTiO_3_: Density functional characterization. AIP Adv..

[38] Humayun M., Xu L., Zhou L., Zheng Z., Fu Q., Luo W. (2018). Exceptional co-catalyst free photocatalytic activities of B and Fe co-doped SrTiO_3_ for CO_2_ conversion and H_2_ evolution. Nano Res..

[39] Modak B., Ghosh S.K. (2015). Exploring the role of La codoping beyond charge compensation for enhanced hydrogen evolution by Rh-SrTiO_3_. J. Phys. Chem. B.

[40] Liu P., Nisar J., Pathak B., Ahuja R. (2012). Hybrid density functional study on SrTiO_3_ for visible light photocatalysis. Int. J. Hydrog. Energy.

[41] Modak B., Srinivasu K., Ghosh S.K. (2014). A hybrid DFT based investigation of the photocatalytic activity of cation-anion codoped SrTiO_3_ for water splitting under visible light. Phys. Chem. Chem. Phys..

[42] Shan J., Raziq F., Humayun M., Zhou W., Qu Y., Wang G., Li Y. (2017). Improved charge separation and surface activation via boron-doped layered polyhedron SrTiO_3_ for co-catalyst free photocatalytic CO_2_ conversion. Appl. Catal. B.

[43] Ohno T., Tsubota T., Nakamura Y., Sayama K. (2005). Preparation of S, C cation-codoped SrTiO_3_ and its photocatalytic activity under visible light. Appl. Catal. A.

[44] Chiang T.H., Lyu H., Hisatomi T., Goto Y., Takata T., Katayama M., Minegishi T., Domen K. (2018). Efficient Photocatalytic Water Splitting Using Al-Doped SrTiO_3_ Coloaded with Molybdenum Oxide and Rhodium–Chromium Oxide. ACS Catal..

[45] Lyu H., Hisatomi T., Goto Y., Yoshida M., Higashi T., Katayama M., Takata T., Minegishi T., Nishiyama H., Yamada T. (2019). An Al-doped SrTiO_3_ photocatalyst maintaining sunlight-driven overall water splitting activity for over 1000 h of constant illumination. Chem. Sci..

[46] Takata T., Jiang J., Sakata Y., Nakabayashi M., Shibata N., Nandal V., Seki K., Hisatomi T., Domen K. (2020). Photocatalytic water splitting with a quantum efficiency of almost unity. Nature.

[47] Wang S., Teramura K., Hisatomi T., Domen K., Asakura H., Hosokawa S., Tanaka T. (2020). Effective Driving of Ag-Loaded and Al-Doped SrTiO_3_ under Irradiation at λ > 300 nm for the Photocatalytic Conversion of CO_2_ by H_2_O. ACS Appl. Energy Mater..

[48] Dawson J.A., Chen H., Tanaka I. (2014). Combined Ab Initio and Interatomic Potentials Based Assessment of the Defect Structure of Mn-Doped SrTiO_3_. J. Phys. Chem. C.

[49] Savinov M., Trepakov V.A., Syrnikov P.P., Železný V., Pokorný J., Dejneka A., Jastrabík L., Galinetto P. (2008). Dielectric properties of Mn doped SrTiO_3_. J. Phys. Condens. Matter.

[50] Azzoni C.B., Mozzati M.C., Paleari A., Massarotti V., Bini M., Capsoni D. (2000). Magnetic evidence of different environments of manganese ions in Mn-substituted strontium titanate. Solid State Commun..

[51] Nomura K., Yamakawa S., Kasari M., Koike Y., Nakanishi A., Kubuki S., Okazawa A. (2019). Magnetic property and Fe Mössbauer analysis of dilute Fe and Nb codoped SrTiO_3-δ_(STO) perovskites. Hyperfine Interact..

[52] Valant M., Kolodiazhnyi T., Arčon I., Aguesse F., Axelsson A.-K., Alford N.M. (2012). The Origin of Magnetism in Mn-Doped SrTiO_3_. Adv. Funct. Mater..

[53] Zorko A., Pregelj M., Luetkens H., Axelsson A.K., Valant M. (2014). Intrinsic paramagnetism and aggregation of manganese dopants in SrTiO_3_. Phys. Rev. B.

[54] Choudhury D., Pal B., Sharma A., Bhat S.V., Sarma D.D. (2013). Magnetization in electron- and Mn-doped SrTiO_3_. Sci. Rep..

[55] Inaba J., Katsufuji T. (2005). Large magnetoresistance in spin- and carrier-doped SrTiO_3_. Phys. Rev. B.

[56] Bannikov V.V., Shein I.R., Kozhevnikov V.L., Ivanovskii A.L. (2008). Magnetism without magnetic ions in non-magnetic perovskites SrTiO_3_, SrZrO_3_ and SrSnO_3_. J. Magn. Magn. Mater..

[57] Guo Y., Qiu X., Dong H., Zhou X. (2015). Trends in non-metal doping of the SrTiO_3_ surface: A hybrid density functional study. Phys. Chem. Chem. Phys..

[58] Liao X.X., Wang H.-Q., Zheng J.-C. (2013). Tuning the Structural, Electronic, and Magnetic Properties of Strontium Titanate through Atomic Design: A Comparison Between Oxygen Vacancies and Nitrogen Doping. J. Am. Ceram. Soc..

[59] Yang K., Dai Y., Huang B. (2012). First-principles characterization of ferromagnetism in N-doped SrTiO_3_ and BaTiO_3_. Appl. Phys. Lett..

[60] Cristiano F., Hebras X., Cherkashin N., Claverie A., Lerch W., Paul S. (2003). Clusters formation in ultralow-energy high-dose boron-implanted silicon. Appl. Phys. Lett..

[61] Yang K., Dai Y., Huang B., Whangbo M.-H. (2009). Density functional studies of the magnetic properties in nitrogen doped TiO_2_. Chem. Phys. Lett..

[62] Kresse G., Furthmuller J. (1996). Efficiency of ab-initio total energy calculations for metals and semiconductors using a plane-wave basis set. Comput. Mater. Sci..

[63] Kresse G., Furthmuller J. (1996). Efficient iterative schemes for ab initio total-energy calculations using a plane-wave basis set. Phys. Rev. B.

[64] Kohn W., Sham L.J. (1965). Self-Consistent Equations Including Exchange and Correlation Effects. Phys. Rev..

[65] Perdew J.P., Burke K., Ernzerhof M. (1996). Generalized Gradient Approximation Made Simple. Phys. Rev. Lett..

[66] Feng N., Wang Q., Zheng A., Zhang Z., Fan J., Liu S.B., Amoureux J.P., Deng F. (2013). Understanding the high photocatalytic activity of (B, Ag)-codoped TiO_2_ under solar-light irradiation with XPS, solid-state NMR, and DFT calculations. J. Am. Chem. Soc..

[67] Cuong D.D., Lee B., Choi K.M., Ahn H.-S., Han S., Lee J. (2007). Oxygen vacancy clustering and electron localization in oxygen-deficient SrTiO_3_: LDA + U study. Phys. Rev. Lett..

[68] Sikam P., Moontragoon P., Sararat C., Karaphun A., Swatsitang E., Pinitsoontorn S., Thongbai P. (2018). DFT calculation and experimental study on structural, optical and magnetic properties of Co-doped SrTiO_3_. Appl. Surf. Sci..

