# Supplementary material for: Tuning the Magnetism in Boron-Doped Strontium Titanate
Source: Materials (Basel). 2020 Dec 12;13(24):5686. doi: 10.3390/ma13245686 (PMC7763848; doi:10.3390/ma13245686)
Supplement: Supplementary file 1 [file materials-13-05686-s001.pdf]

materials

MDPI

Supplemental

Tuning the Magnetism in Boron-Doped Strontium Titanate

Hui Zeng [1], Meng Wu [1], Hui-Qiong Wang [1,2,*], Jin-Cheng Zheng [1,2,*] and Junyong Kang [1]

[1] Fujian Provincial Key Laboratory of Semiconductors and Applications, Collaborative Innovation Center for Optoelectronic Semiconductors and Efficient Devices, Department of Physics, Xiamen University, Xiamen 361005, China; 19820170155498@stu.xmu.edu.cn (H.Z.); meng.wu@xmu.edu.cn (M.W.); jykang@xmu.edu.cn (J.K.)

[2] Department of Physics, Xiamen University Malaysia, Sepang 43900, Selangor, Malaysia

* Correspondence: hqwang@xmu.edu.cn (H.-Q.W.); jczheng@xmu.edu.cn (J.-C.Z.)

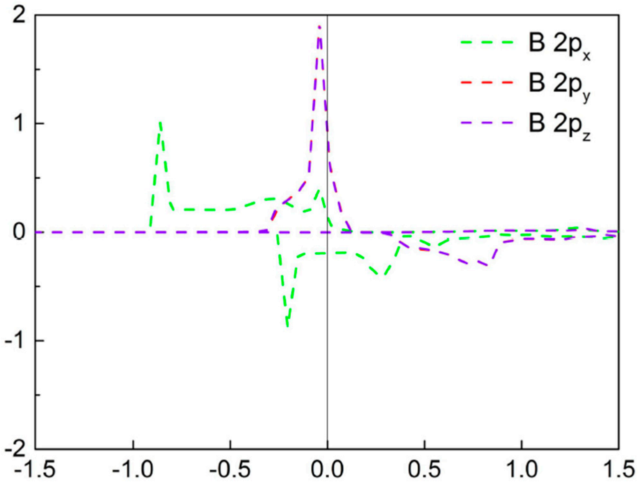

Figure S1. The calculated partial density of states (PDOS) of the B 2p$_x$, 2p$_y$, and 2p$_z$ states in B-doped $SrTiO_3$ (one boron in 40-atom supercell).

Table S1. Average contribution of MM for O (MM_O) and Sr (MM_{Sr}) atom in units of μ_B under FM and AFM alignments for different (0,j) structures in $SrTiO_{2.75}B_{0.25}$.

(0,j)	MM_O(FM/AFM)	MM_{Sr}(FM/AFM)
(0,1)	0/0	0/0
(0,2)	0.03/0	0.02/0
(0,3)	0.02/0	0/0
(0,4)	0.01/0	0.02/0
(0,5)	0.02/0	0.01/0
(0,6)	0.02/0	0.02/0
(0,7)	0.03/0	0.02/0

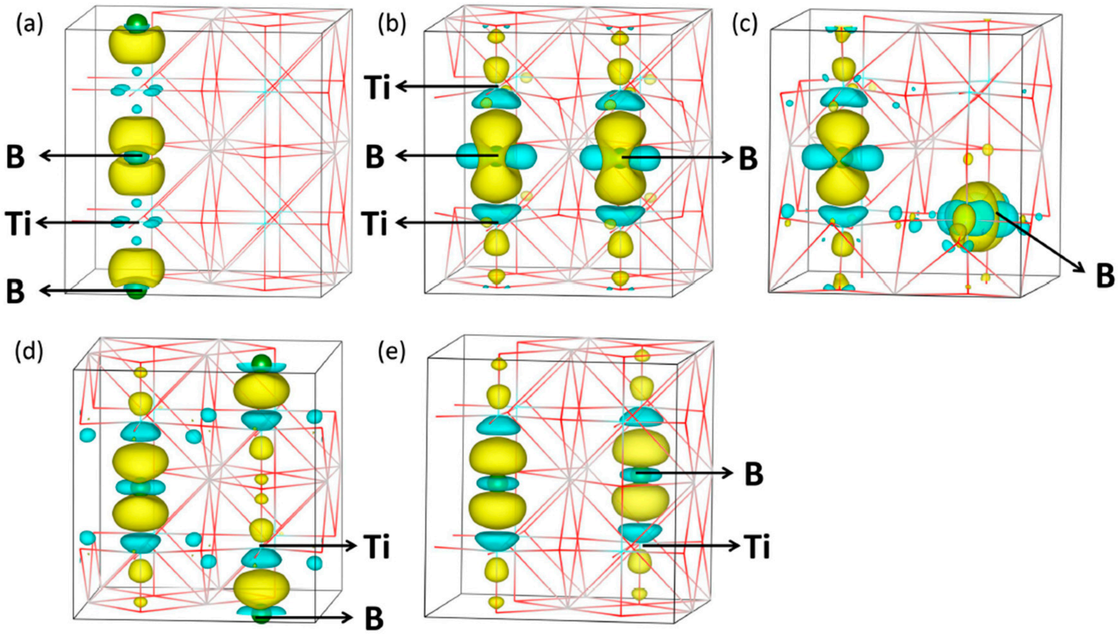

Figure S2. (**a**)–(**e**): The charge density differences from structure (0,2) to (0,6). The yellow and violet red areas represent charge accumulation and charge depletion, respectively. The isosurface level value is set as 0.005 e/Å^3.
